# Effect of spraying *Bacillus coagulans* during incubation on hatchability, chick growth, resistance to *Mycoplasma synoviae*, and cecal microbiota

**DOI:** 10.1016/j.psj.2026.107154

**Published:** 2026-05-20

**Authors:** Yimin Wei, Xuli Zhao, Xing Chen, Xiaomeng Miao, Wen Li, Ying Ma, Xiaoyu Zhao, Zhonghua Ning

**Affiliations:** a National Engineering Laboratory for Animal Breeding, College of Animal Science and Technology, China Agricultural University, Beijing 100193, China; b Baoding Xingrui Agriculture and Animal Husbandry Development Co., Ltd, Baoding 071001, China; c Institute of Animal Husbandry and Veterinary Medicine, Guizhou Academy of Agricultural Sciences, Guiyang 550005, China

**Keywords:** *Bacillus coagulans*, Early probiotic colonization, *Mycoplasma synoviae* infection, Spray method

## Abstract

*Mycoplasma synoviae* (**MS**) causes synovitis and airsacculitis in laying hens, reducing production performance and resulting in economic losses. Leveraging the acid-intolerant nature of MS, this study sprayed acid-producing *Bacillus coagulans* (***B. coagulans***) during incubation, investigating the effects on hatchability, chick growth, resistance to MS, and cecal microbiota. For this study, hatching eggs in the treatment group were sprayed with either *B. coagulans* or saline, with a conventional incubation group serving as the control. Results indicated that the *B. coagulans* sprayed group exhibited a higher percentage of healthy day-old chicks compared to other groups (*P* < 0.01). Post-hatch, chicks were divided into five groups based on treatments during incubation and post-hatch: Group A (sprayed with *B. coagulans* + provided drinking water), Group B (sprayed with sterile saline + fed with *B. coagulans)*, Group C (sprayed with sterile saline + provided drinking water), Group D (sprayed with sterile saline + fed with antibiotics), Group E (sprayed with *B. coagulans* + fed with antibiotics). Results indicated that Group A demonstrated superior growth performance during the first week. At 21 days of age, Group A showed a lower MS infection rate compared to all other groups (*P* < 0.01), while serum levels of IL-10, IgA, and IgG were upregulated in Groups A and E (*P* < 0.01). 16S rRNA sequencing analysis revealed that *B. coagulans* spray treatment improved cecal microbiota homogeneity and increased the relative abundance of Eubacterium coprostanoligenes (**E. coprostanoligenes**) and *Negativibacillus* (*P* < 0.05), promoting enrichment of short-chain fatty acid-producing genera. Further analysis revealed a positive correlation between the relative abundance of E. coprostanoligenes and MS resistance, suggesting its colonization advantage may directly enhance host immunity against MS. In summary, spraying *B. coagulans* during incubation promoted hatchability, chick growth, resistance to MS, and cecal microbiota. This strategy provides an antibiotic-free solution for MS control, with operational simplicity and sustained efficacy that align with the demands of modern intensive poultry farming.

## Introduction

*Mycoplasma synoviae* (**MS**) is an important pathogenic agent in poultry, belonging to the genus *Mycoplasma* within the class Mollicutes. Infected chickens exhibit arthritis and upper respiratory tract lesions (Zhao et al., 2025). Once a flock becomes infected, eradication of the pathogen is notably challenging, and infected chickens typically remain carriers for life. Due to its chronic nature, MS is responsible for substantial economic losses in the poultry industry worldwide. Research indicates that egg production and hatchability decrease by 5–10% following infection (Yadav et al., 2021). It can also reduce egg quality parameters and result in eggshell apex abnormalities (Wang et al., 2025). How to prevent MS in a cost-effective way has become an urgent issue in poultry production.

Compared to chicks in contact with adult hens, commercially hatched chicks have a higher hatch rate but a lower gut microbiota diversity index (Rychlik et al., 2020); when pathogens are present in the environment, commercially hatched chicks are more susceptible to infection. Probiotics can regulate the gut microbiota through various mechanisms, thereby boosting immunity and improving poultry production performance (Jha et al., 2020). The spray method is a technique that facilitates the early colonization of probiotics during incubation. This method uses atomization equipment to evenly spray the probiotic suspension onto eggshells during incubation. It accomplishes early probiotic colonization by taking advantage of the physiological behavior of chicks pecking through the eggshell before hatching. Moreover, spraying is a standard pre-hatching disinfection method (Melo et al., 2019), and large-scale poultry farms are commonly equipped with the necessary apparatus, making this approach cost-effective and straightforward to implement.

*Bacillus coagulans* (***B. coagulans***) not only produces acid, like lactic acid bacteria, to inhibit the growth and reproduction of harmful microorganisms, but also forms spore structures that can withstand temperatures up to 90 °C (Mazhar et al., 2024), maintaining viability under conventional incubator temperatures. Additionally, through mechanisms like interference with flagellar protein synthesis, the antimicrobial peptides generated by its metabolism effectively prevent the growth of pathogenic bacteria like *Salmonella* (Sharifi et al., 2021). *B. coagulans* can pass through the stomach and enter the intestines due to its ability to withstand bile and gastric acid (Mazhar et al., 2024). Multiple studies confirm that *B. coagulans* enhances intestinal health and production performance in the host by improving intestinal mucosal morphology, modulating microbial homeostasis, and boosting immune responses (Liu et al., 2022; Niu et al., 2025; Zhang et al., 2021).

Based on the considerable threat that MS poses to poultry production, this study investigates the effects of early probiotic colonization by spraying *B. coagulans* during incubation on hatchability, chick growth, resistance to MS, and cecal microbiota. Aiming to develop an early probiotic colonization technique that is operational simplicity, sustained efficacy, and suitable for modern intensive poultry farming, thereby providing a new solution for achieving antibiotic-free poultry farming and enhancing the quality and safety of poultry products.

## Materials and methods

### Ethics statement

The study was conducted in accordance with the guidelines for experimental animals established by the Animal Care and Use Committee of China Agricultural University, China. The approval number is: Aw81506202-01-01.

### Experimental animals and experimental design

A total of 7,129 fertilized eggs from 28-week-old Dawu JinFeng laying hens were used in the incubation experiment and randomly assigned to three groups: *B. coagulans* spray group (*n* = 2,348), sterile saline spray group (*n* = 2,345), and conventional incubation control group (*n* = 2,436). All eggs were confirmed fertilized through candling. For the *B. coagulans* spray group, the bacterial suspension was sprayed onto the egg surfaces on day 18 of incubation. The sterile saline spray group received an equivalent volume of sterile saline spray, while the conventional incubation group received no treatment.

After hatching, healthy chicks (free of MS infection) from the *B. coagulans* spray group and the sterile saline spray group were selected for growth performance, MS disease resistance, and cecal microbiota comparison experiment. They were divided into five groups based on treatments during incubation and post-hatch: Group A (sprayed with *B. coagulans* + provided drinking water), Group B (sprayed with sterile saline + fed with *B. coagulans)*, Group C (sprayed with sterile saline + provided drinking water), Group D (sprayed with sterile saline + fed with antibiotics), Group E (sprayed with *B. coagulans* + fed with antibiotics), with 12 replicates of 14 broilers each. All chicks were reared in an MS-contaminated environment (after the culling of the previous batch of chickens infected with MS, the henhouse was not disinfected).

The spray concentration of *B. coagulans* was 4 × 10⁶ CFU/mL, prepared by dissolving 6.00 g of *B. coagulans* solid powder (Kunming Sanzheng Biotechnology Co., Ltd, Yunnan, CN) in 300 mL sterile saline to form a bacterial suspension. Antibiotics were doxycycline hydrochloride and tylosin (Shandong Luxi Veterinary Medicine Co., Ltd, Shandong, CN). For administration, 1.5 g of 45% tylosin soluble powder and 1.5 g of 10% doxycycline hydrochloride soluble powder were added to 6 L of drinking water and mixed thoroughly.

### Reproductive performance and growth performance

Percentage of healthy day-old chicks, Hatchability of settable eggs, and Embryonic mortality rate were calculated using the following formulas:

$$\text{Percentage}\;\text{of}\;\text{healthy}\;\text{day}-\text{old}\;\text{chicks}\;(\%)\;=\;\frac{\text{number}\;\text{of}\;\text{healthy}\;\text{day}\;\text{old}\;\text{chicks}}{\text{number}\;\text{of}\;\text{chicks}\;\text{hatched}}\;\times \;100$$

$$\text{Hatchability}\;\text{of}\;\text{fertilized}\;\text{eggs}\;(\%)\;=\;\frac{\text{number}\;\text{of}\;\text{chicks}\;\text{hatched}}{\text{number}\;\text{of}\;\text{fertilized}\;\text{eggs}}\;\times \;100$$

$$\text{Embryonic}\;\text{mortality}\;\text{rate}\;(\%)\;=\;\frac{\text{number}\;\text{of}\;\text{unhatched}\;\text{eggs}\;+\;\text{number}\;\text{of}\;\text{dead}\;\text{embryos}}{\text{number}\;\text{of}\;\text{fertilized}\;\text{eggs}}\;\times \;100$$

BW was recorded on days 1, 7, 14, and 21 post-hatch by weighing all chicks within each replicate. ADG was calculated using the following formula:

$$\text{ADG}\;(g/d)\;=\;\frac{\text{final}\;\text{BW}\;-\;\text{initial}\;\text{BW}}{\text{number}\;\text{of}\;\text{days}}$$

### MS infection rate and serum immune indices

Three chicks were randomly selected from each cage on days 1, 7, 14, 21, and 28 post-hatch. Throat swabs were collected for DNA extraction, followed by qPCR testing (qPCR primer and probe sequences are shown in Table 1) to determine the MS infection rate. qPCR System: TransStart Probe qPCR SuperMix (2 ×) 10 μL, upstream and downstream primers 0.4 μL each, probe 0.4 μL, DNA template 2 μL, ddH_2_O 6.8 μL. qPCR Protocol: 95°C pre-denaturation for 30 s; 95°C denaturation for 5 s; 60°C annealing for 60 s, repeated for a total of 40 cycles. Results are determined based on Ct values.

**Table 1:** Primer and probe sequences for qPCR.

Primer name	Probe sequences (5′−3′)
MS-vlhA-FAM	AGCAATTTCATGTGGTGATMAAACTCCA
MS-F	TTRCTATTAGCAGCTAGTGCAGTGGC
MS-R	AGGATTATCAGTATTTGGGTTTCCAG

MS denotes *Mycoplasma synoviae*.

Five uninfected chicks were randomly selected from each group for serum immune indices analysis on day 21 post-hatch. Blood was collected via the jugular vein, and serum was separated. Following the standard operating procedures of the kit (Sierai Bio, Beijing, CN), the levels of IL-1β, IL-6, IL-10, IgA, IgG, and IgM were measured.

### Cecal microbiota

On day 21 post-hatch, five uninfected chickens were randomly selected from each group for cecal microbial analysis. Chicks were sacrificed by cervical dislocation. Microbial genomic DNA was extracted from cecal chyme samples using TIANGEN Tissue Genomic DNA Extraction Kit (Tiangen Biochemical Technology Co., Ltd, Beijing, CN). Agarose gel electrophoresis was performed to measure DNA concentration and purity. The bacterial 16S rRNA gene targeted V1-V9 variable region was amplified through PCR. PCR amplification was performed as follows: 98°C denaturation for 150 s; 50°C annealing for 30 s;72°C extension for 360 s, repeated for a total of 25 cycles. The amplification products were sequenced on the Illumina MiSeq PE300 platform (Illumina, San Diego, CA, USA).

Raw reads were filtered using Trimmomatic v0.39, and primer sequences were removed using cutadapt v3.5 to generate clean reads. Operational taxonomic units (**OTUs**) were generated by clustering sequences at 97% similarity with Uparse v7.0.1090, from which representative sequences were selected. Alpha diversity was evaluated using richness indices (ACE and Chao1) and diversity indices (Shannon and Simpson). Beta diversity was assessed based on principal coordinate analysis (**PCoA**) using Bray–Curtis distances. Differences in microbial community structure were further evaluated using analysis of similarities (Anosim). Key bacterial taxa were identified through linear discriminant analysis (**LDA**) effect size (**LEfSe**). Taxa with an LDA > 2 and *P* < 0.05 were regarded as significant. Differences between groups were evaluated using the Kruskal–Wallis H test. Graphical visualization was performed using the R v3.3.1 software.

### Statistical analysis

Experimental data on reproductive performance, growth performance, MS infection rate, and serum immune indices were first normalized and organized using Microsoft Excel. Subsequently, SPSS 26.0 was employed to conduct significance tests on the experimental data. Differences were determined to be significant at *P* < 0.05 and highly significant at *P* < 0.01. Since the dependent variable is binary categorical data and the sample size in each replicated treatment group is greater than 40, the chi-square test was employed for data analysis. For the analysis of MS positivity rates, where the sample size in each treatment group was less than 40, and there were expected frequencies of less than 1, Fisher's exact test was used for data processing and analysis.

## Results

### Reproductive performance and growth performance

Table 2 shows the effects of the spray method on reproductive performance. The Percentage of healthy day-old chicks in the *B. coagulans* spray group is higher than the other two groups (*P* < 0.01). Differences in Hatchability of fertilized eggs and Embryonic mortality rate did not reach statistical significance. However, compared to the other two groups, the *B. coagulans* spray group exhibited numerically higher Hatchability of fertilized eggs and lower Embryonic mortality rate. This indicates that the spray method can improve reproductive performance.

**Table 2:** Effects of different treatments on reproductive performance in chicks.

Testing indicators	Treatment			*P* value
	probiotic	saline	control	
Percentage of healthy day-old chicks (%)	95.92^A^	93.60^B^	93.53^B^	< 0.01
Hatchability of settable eggs (%)	97.15	96.67	97.09	0.59
Embryonic mortality rate (%)	2.85	3.33	2.91	0.59

A-B The values in the same row with different superscript letters differ statistically (*P* < 0.01).

Effects of different treatments on growth performance are shown in Table 3. Group A exhibited the highest BW and ADG during the first week post-hatch, but there were no differences compared to the other groups, except Group B. Furthermore, no differences in BW or ADG were observed between groups during the second and third weeks post-hatch, suggesting that the growth-promoting effect of the spray treatment was most evident during the early post-hatch period and gradually diminished as chicks adapted to the rearing environment.

**Table 3:** Effects of different treatments on growth performance in chicks.

Testing indicators	Treatment					*P* value
	A	B	C	D	E	
BW (g)						
1d	36.66 ± 0.43	36.53 ± 0.24	36.61 ± 0.16	36.74 ± 0.23	36.60 ± 0.20	0.50
7d	75.66 ± 1.25^a^	74.00 ± 1.49^b^	74.55 ± 0.86^ab^	75.06 ± 0.90^a^^b^	74.76 ± 1.59^ab^	0.04
14d	134. 14 ± 4.23	133.54 ± 2.52	135.27 ± 2.45	133.68 ± 3.21	134.35 ± 3.14	0.55
21d	211.53 ± 6.31	210.97 ± 4.73	212.97 ± 6.00	213.10 ± 5.36	212.25 ± 6.07	0.88
ADG (g/d)						
1-7d	5.57 ± 0.18	5.35 ± 0.20	5.42 ± 0.14	5.48 ± 0.15	5.45 ± 0.21	0.07
8-14d	8.40 ± 0.47	8.50 ± 0.27	8.67 ± 0.35	8.37 ± 0.39	8.65 ± 0.26	0.19
15-21d	11.01 ± 0.97	11.06 ± 0.56	11.10 ± 0.65	11.34 ± 1.03	11.11 ± 0.42	0.88

Data are presented as mean ± SEM.

a-b The values in the same row with different superscript letters differ statistically (*P* < 0.05). BW denotes body weight, and ADG denotes average daily gain. Group A: sprayed with *Bacillus coagulans (B. coagulans)* + provided drinking water, Group B: sprayed with sterile saline + fed with *B. coagulans*, Group C: sprayed with sterile saline + provided drinking water, Group D: sprayed with sterile saline + fed with antibiotics, Group E: sprayed with *B. coagulans* + fed with antibiotics.

### MS infection rate and serum immune indices

Table 4 indicates that the MS infection rate increased progressively across different treatment groups as the feeding time extended. On 7 days post-hatch, MS-infected individuals were detected in Groups C and D. On 14 days post-hatch, only Group A remained free of MS infection. On 21 days post-hatch, all treatment groups contained MS-infected individuals, and Group A showed the lowest MS infection rate. On the 28 days post-hatch, the MS infection rate in Groups A and E (groups that used the spray method) was lower than in Groups B, C, and D (*P* < 0.01). These findings indicate that spray *B. coagulans* during incubation is more effectively at enhancing resistance to MS than feeding *B. coagulans* or using antibiotic after hatching.

**Table 4:** MS infection rate in different treatment groups of chicks.

Testing indicators	Treatment					*P* value
	A	B	C	D	E	
Positivity rate (%)						
1d	0.00	0.00	0.00	0.00	0.00	1.00
7d	0.00	0.00	5.56	2.78	0.00	0.51
14d	0.00	13.89	8.33	2.78	5.56	0.15
21d	2.78^C^	50.00^A^	44.44^A^^B^	56.56^A^	16.67^BC^	< 0.01
28d	11.11^B^	86.11^A^	80.56^A^	86.11^A^	38.89^B^	< 0.01

A-B The values in the same row with different superscript letters differ statistically (*P* < 0.01). MS denotes *Mycoplasma synoviae.* Group A: sprayed with *Bacillus coagulans (B. coagulans)* + provided drinking water, Group B: sprayed with sterile saline + fed with *B. coagulans*, Group C: sprayed with sterile saline + provided drinking water, Group D: sprayed with sterile saline + fed with antibiotics, Group E: sprayed with *B. coagulans* + fed with antibiotics.

Analysis of serum immune indices of different treatment groups at 21 days post-hatch is shown in Table 5. On 21 days post-hatch, IL-1β and IL-6 levels were lower in Groups D and E, with the highest concentrations in Group B, indicating that post-hatch *B. coagulans* feeding was associated with stronger pro-inflammatory responses. IL-10 levels in Groups A, D, and E were higher than in Groups B and C(*P* < 0.01), suggesting enhanced anti-inflammatory regulation in the spray-treated or antibiotic-treated groups. IgA and IgG levels were higher in Groups A and E than in Groups B, C, and D, indicating that *B. coagulans* spraying during incubation may strengthen mucosal and humoral immune responses. IgM levels showed no differences among groups, suggesting that IgM was not the main immune factor associated with the observed differences in MS resistance at this stage.

**Table 5:** Analysis of serum immune indices of different treatment groups at 21 days of age.

Testing indicators	Treatment					*P* value
	A	B	C	D	E	
IL-1β (ng/L)	93.67 ± 6.92^B^	124.15 ± 8.47^A^	105.54 ± 0.46^A^^B^	61.29 ± 0.25^C^	87.04 ± 20.63^B^	< 0.01
IL-6 (ng/L)	31.38 ± 3.54^a^	31.78 ± 5.30^a^	24.02 ± 0.04^ab^	22.03 ± 0.05^b^	24.76 ± 6.80^ab^	0.04
IL-10 (ng/L)	50.48 ± 1.45^A^	36.38 ± 9.30^B^	36.16 ± 0.14^B^	50.27 ± 0.24^A^	50.92 ± 2.04^A^	< 0.01
IgA (ng/mL)	8798.2 ± 0.18^A^	7362.56 ± 1366.63^B^	4886.07 ± 0.06^C^	6367.04 ± 360.27^B^	8567.12 ± 760.60^A^	< 0.01
IgG (μg/mL)	70.2 ± 0.17^AB^	39.05 ± 14.34^C^	45.67 ± 6.25^C^	59.41 ± 0.35^B^	75.09 ± 8.51^A^	< 0.01
IgM (ng/mL)	5449.73 ± 397.23	5352.42 ± 477.06	5158.34 ± 0.30	5228.62 ± 0.54	4866.61 ± 544.52	0.27

Data are presented as mean ± SEM.

A-B The values in the same row with different superscript letters differ statistically (*P* < 0.01). Group A: sprayed with *Bacillus coagulans (B. coagulans)* + provided drinking water, Group B: sprayed with sterile saline + fed with *B. coagulans*, Group C: sprayed with sterile saline + provided drinking water, Group D: sprayed with sterile saline + fed with antibiotics, Group E: sprayed with *B. coagulans* + fed with antibiotics.

### Cecal microbiota

**Fig. 1:**
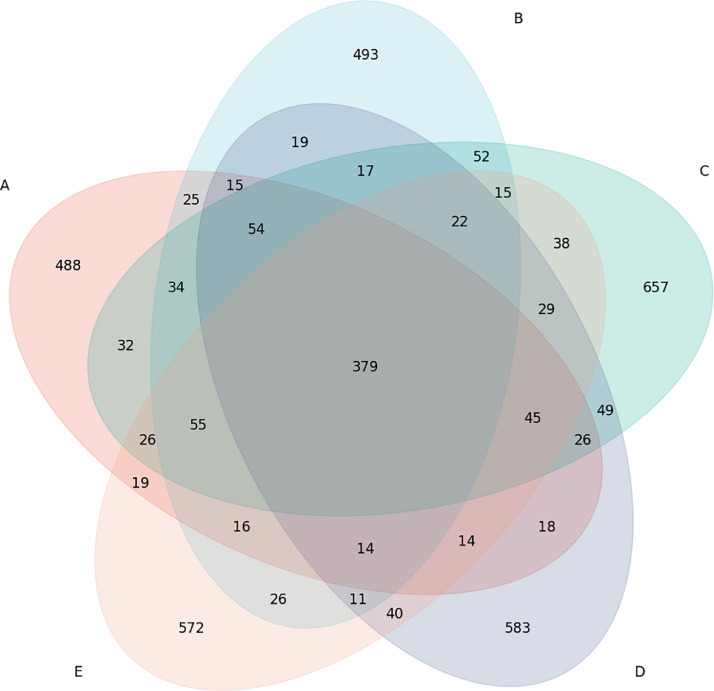
Effects of different treatments on cecal microbiota OTUs. Group A: sprayed with *Bacillus coagulans (B. coagulans)* + provided drinking water, Group B: sprayed with sterile saline + fed with *B. coagulans*, Group C: sprayed with sterile saline + provided drinking water, Group D: sprayed with sterile saline + fed with antibiotics, Group E: sprayed with *B. coagulans* + fed with antibiotics.

After sequencing, the data from each group were analyzed. Groups A, B, C, D, and E exhibited 488, 493, 657, 583, and 572 unique OTUs, respectively (Fig. 1).

**Fig. 2:**
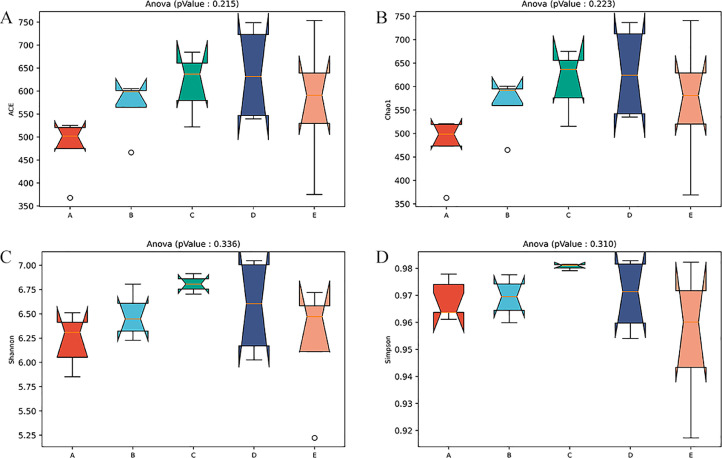
Effects of different treatments on alpha diversity of cecal microbiota. (A) ACE index. (B) Chao1 index. (C) Shannon index. (D) Simpson index. Group A: sprayed with *Bacillus coagulans (B. coagulans)* + provided drinking water, Group B: sprayed with sterile saline + fed with *B. coagulans*, Group C: sprayed with sterile saline + provided drinking water, Group D: sprayed with sterile saline + fed with antibiotics, Group E: sprayed with *B. coagulans* + fed with antibiotics.

Alpha diversity primarily reflects the richness and evenness of intestinal flora. The results of alpha diversity analysis in Fig. 2 indicate that there are no differences in the ACE, Chao1, Shannon, and Simpson indices among groups.

**Fig. 3:**
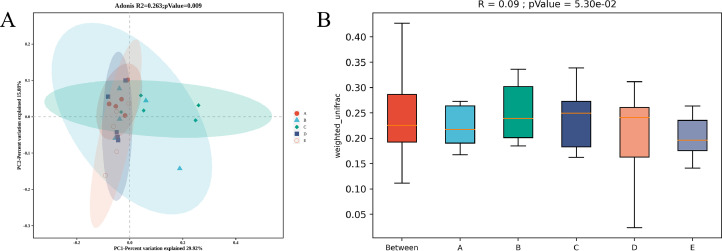
Effects of different treatment groups on Beta diversity of cecal microbiota. (A) PCoA Plot. (B) Anosim Plot. Group A: sprayed with *Bacillus coagulans (B. coagulans)* + provided drinking water, Group B: sprayed with sterile saline + fed with *B. coagulans*, Group C: sprayed with sterile saline + provided drinking water, Group D: sprayed with sterile saline + fed with antibiotics, Group E: sprayed with *B. coagulans* + fed with antibiotics.

The PCoA plot in Fig. 3 reveals that Group A exhibits the most concentrated microbial community distribution, indicating greater homogeneity in its microbial community structure. The confidence ellipses of Group E nearly overlapped with Groups A and D, indicating similarities among the microbial communities in Groups A, D, and E. The confidence ellipses of Groups B and C were distinctly separated from Groups A, E, and D, but the anosim analysis reveals no differences between or within groups.

Firmicutes, Bacteroidetes, Actinobacteriota, and Proteobacteria were the dominant phyla of the cecum in all treatments. No significant difference in the relative abundance of cecal microbiota communities was found among different treatment groups at the phylum level (results not shown).

**Fig. 4:**
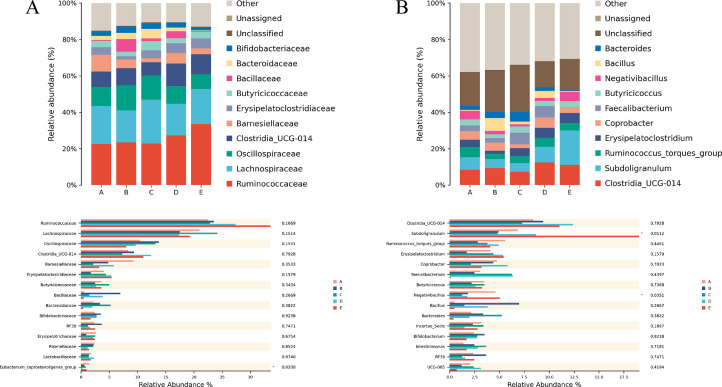
Relative abundance of the cecal microbiota under different treatments. (A) Family-level. (B) Genus-level. Group A: sprayed with *Bacillus coagulans (B. coagulans)* + provided drinking water, Group B: sprayed with sterile saline + fed with *B. coagulans*, Group C: sprayed with sterile saline + provided drinking water, Group D: sprayed with sterile saline + fed with antibiotics, Group E: sprayed with *B. coagulans* + fed with antibiotics.

Relative abundance of cecal microbiota at the family and genus levels are shown in Fig. 4 There was a difference of the relative abundance of Eubacterium coprostanoligenes (**E. coprostanoligenes**) among treatment groups at the family level (*P* < 0.05), with relative abundance ranking as Groups *A* > *E* > *C* > *B* > *D*. This trend highly correlated with the MS disease resistance levels across Groups *A* > *E* > *C* > *B* ≥ *D*.

At the genus level, there were differences in the relative abundance of *Subdoligranulum* and *Negativibacillus* among treatment groups (*P* < 0.05). The relative abundance of *Subdoligranulum* was higher in Groups D and E (antibiotic-treated groups). The relative abundance of *Negativibacillus* was higher in Groups A and E (spray-treated groups).

**Fig. 5:**
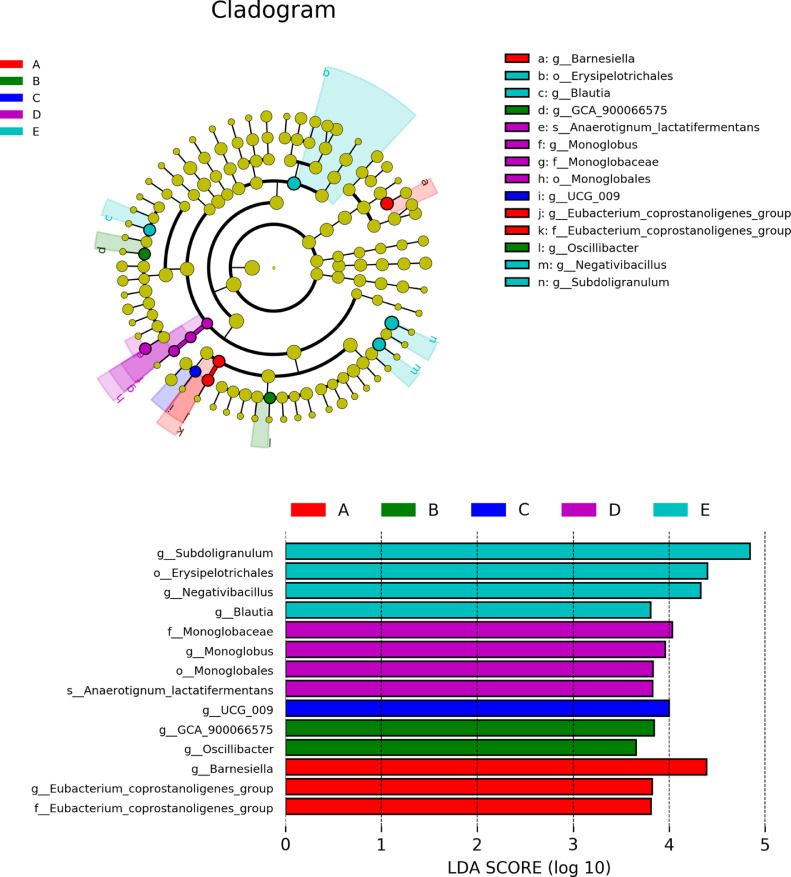
Differentially abundant taxa among treatment groups identified by linear discriminant analysis effect size. Group A: sprayed with *Bacillus coagulans (B. coagulans)* + provided drinking water, Group B: sprayed with sterile saline + fed with *B. coagulans*, Group C: sprayed with sterile saline + provided drinking water, Group D: sprayed with sterile saline + fed with antibiotics, Group E: sprayed with *B. coagulans* + fed with antibiotics.

The results of LEfSe analysis in Fig. 5 indicate that microbial communities across different experimental groups exhibit specific enrichment patterns. In Group A, the relative abundance of genera such as *f_Eubacterium_coprostanoligenes_group, g_Barnesiella*, and *g_Eubacterium_coprostanoligenes_group* significantly increased; Group B's dominant microbiota centered on *g_GCA_900066575* and *g_Oscillibactero*; Group C's dominant genus was *g_UCG_009*; Group D exhibited a pronounced enrichment of Monoglobales microorganisms (including *o_Monoglobales, f_Monoglobaceae, g_Monoglobus*), besides *s_Anaerotignum_lactatifermentans* also enriched in this group; In Group E, the relative abundances of the genera *Blautia, Negativibacillus*, and *Subdoligranulum*, along with *o_Erysipelotrichaceae*, were significantly higher than in other groups. The core feature shared by Group A and Group E is the application of *B. coagulans* spray, with both groups exhibiting synergistic enrichment of cholesterol-metabolizing bacteria such as *E. coprostanoligenes* and short-chain fatty acid (**SCFA**)-producing bacteria such as *Subdoligranulum, Blautia* in their microbial communities.

## Discussion

### Reproductive performance and growth performance

According to statistical analysis, spraying *B. coagulans* onto eggs during incubation improves the percentage of healthy day-old chicks. This phenomenon may be due to *B. coagulans* adhering to the eggshell and forming a biofilm (Midhun et al., 2018). As chicks peck through the shell, *B. coagulans* enters their digestive tract via the mouth and rapidly colonizes the intestines. Leveraging its metabolic characteristics and competitive advantages (Zhu et al., 2023), the probiotic occupies an ecological niche, enabling beneficial bacteria to dominate the gut microbiota. This transformation optimizes the intestinal microecology, improves intestinal barrier function and immune defense capabilities, reduces the risk of harmful microbial invasion, and consequently improves the percentage of healthy day-old chicks.

The growth advantage observed in Group A during the first week may be attributable to the spray method facilitating early probiotic colonization. Research indicates that the spraying method allows direct contact of probiotics with the gastrointestinal and respiratory tract mucosa of chicks, thereby promoting rapid colonization in vivo (Muyyarikkandy et al., 2023). The absence of differences among groups in the second and third weeks may be associated with the physiological adaptation of chicks and the establishment of microbial homeostasis. As the chicks' endogenous microbiota stabilized, the marginal benefit of exogenous supplementation diminished. Furthermore, the disappearance of statistical differences does not imply a lack of biological relevance. The weight gain in the first week may lay the foundation for subsequent growth and development, such as enhancing skeletal development potential or immune function (Lamot, 2017).

### MS infection rate and serum immune indices

All groups showed an increasing trend in MS infection rates at 14 days post-hatch, which may be related to the depletion of maternal antibodies (Woziri et al., 2021). By 21 days post-hatch, Group A showed the lowest infection rate, followed by Group E, suggesting that spray probiotics can enhance immune function. Combined with the results of serum immune indices, this may be due to the spray method enabling early direct contact between the gastrointestinal and respiratory tract mucosa with *B. coagulans*, thereby activating the phagocytic activity of local immune cells and promoting IgA production, forming a mucosal barrier to block MS colonization (Zhang et al., 2021). Group E exhibited a lower infection rate than Group D but higher than Group A. This suggests that antibiotics may partially inhibit *B. coagulans* activity. Conversely, *B. coagulans* may partially counteract antibiotic suppression of the immune system by regulating gut microbiota balance and enhancing the development of immune organs, thereby maintaining anti-infective capacity (Jensen et al., 2017). Although Group D exhibited a lower infection rate during the first 14 days, its infection rate surged in the later stages, which may be related to the immunosuppression caused by antibiotics (Bigelow et al., 2023).

Group B exhibited the highest levels of IL-1β and IL-6, while IL-10 levels were the lowest, suggesting that feeding *B. coagulans* may amplify inflammatory responses through gut immune activation (Shadnoush et al., 2018) without effectively inducing anti-inflammatory factor release. Conversely, Groups A and E maintained elevated IL-10 levels through *B. coagulans* spray application, suggesting that spray administration during the incubation period may be more advantageous for suppressing excessive inflammation. Group D and E exhibited the lower IL-1β and IL-6 levels, while higher levels of IL-10, indicating that antibiotics may possess dual immunomodulatory effects: reducing pro-inflammatory factor expression while promoting anti-inflammatory factor expression (Solis et al., 2018). However, IgA and IgG levels in Group D were lower than in Groups A and E, suggesting that its immunosuppressive effect may limit the production of specific antibodies, leading to uncontrolled infection in later stages.

Groups A and E showed higher IgA levels than other groups; this is consistent with previous reports that *B. coagulans* promotes IgA secretion by activating intestinal mucosal lymphoid tissue (Xu et al., 2017). Elevated IgA levels may block MS adhesion to the mucosa, explaining the low infection rate. Group E exhibited the highest IgG levels, suggesting that combined *B. coagulans* spraying and antibiotic feeding may enhance specific immune responses against MS by promoting B cell differentiation (Chen et al., 2018). No differences in IgM levels were observed across groups, indicating that it was no longer a dominant immune factor at this point.

### Cecal microbiota

Group A exhibited the lowest values for the ACE index, Chao1 index, and Shannon index, which may be attributed to *B. coagulans* inhibiting the proliferation of certain strains by secreting antimicrobial substances such as lactic acid (Oh et al., 2016).

Based on the results of Beta diversity analysis, Group A exhibited the highest sample point clustering, indicating greater homogeneity in its microbial community structure. This aligns with Group A having the lowest number of OTUs. This phenomenon may be related to the early colonization of *B. coagulans, which* inhibits the proliferation of other microbial communities (Zhang et al., 2021). The confidence ellipse of Group B samples deviated from those of Groups A and E, suggesting that gut microbiota composition may be disrupted by the method of probiotic administration. The high overlap between Groups A and E's confidence ellipses may result from probiotic-antibiotic interactions, where *B. coagulans* partially counteracts antibiotic disruption of the microbiota (Dey et al., 2024), leading to similar microbial structures in both groups.

The results of family-level analysis suggest a potential positive correlation between this bacterial group's relative abundance and host disease resistance. Other research indicate that the E. coprostanoligenes plays a crucial role in nutrient metabolism and maintaining intestinal homeostasis, it positively influences metabolic and immune functions by converting cholesterol, producing short-chain fatty acids, and enhancing intestinal barrier function (Li et al., 2024; Ashraf et al., 2024; Deng et al., 2023). Notably, as a mycoplasma, MS requires cholesterol for growth and reproduction. Therefore, spraying *B. coagulans* likely reduces MS infection rates by increasing the relative abundance of the E. coprostanoligenes.

At the genus level, the relative abundance of *Subdoligranulum* was higher in Groups D and E (antibiotic-treated groups). This finding contradicts a previous study, which reported that “antibiotic use reduces *Subdoligranulum* abundance in the human gut” (Wang et al., 2021). This discrepancy may be related to factors such as the type of antibiotic used and host individual differences, such as variations in gut ecology between poultry and humans. *Negativibacillus* showed an enrichment trend in both Group A and Group E. *Negativibacillus* belongs to the *Ruminococcaceae* family and remains understudied. However, given its isolation from healthy human intestines (Ricaboni et al., 2016) and the fact that *Ruminococcaceae* family members typically participate in short-chain fatty acid synthesis (Guan et al., 2021), it is speculated that it may exert probiotic effects by maintaining gut microbiota balance or regulating the immune system.

Group A and Group E exhibited a synergistic enrichment of cholesterol-metabolizing bacteria and SCFA-producing bacteria in their microbial communities. This combination may inhibit MS infection through dual pathways: (1) Cholesterol metabolism: MS lacks a cell wall, and its cell membrane construction is highly dependent on cholesterol supplied by the host (Sprankel et al., 2023). *E. coprostanoligenes* converts intestinal cholesterol into coprostanol (Kenny et al., 2020), which itself exhibits antimicrobial properties. Thus, this process not only reduces host cholesterol levels but may also directly disrupt the survival microenvironment of MS pathogens. (2) SCFA-mediated barrier enhancement: *Subdoligranulum* and *Blautia* are major producers of butyrate and acetate, respectively. Butyrate, as the primary energy substrate for intestinal epithelial cells, activates the AMPK signaling pathway to promote the assembly and expression of tight junction proteins (e.g., ZO-1, Occludin), thereby enhancing the integrity of the mucosal physical barrier (Van Hul et al., 2020). while acetate promotes regulatory T cell differentiation, balancing immune responses (Daien et al., 2021). Additionally, SCFAs lower the intestinal pH, creating an acidic microenvironment. Since MS is acid-intolerant, such conditions directly inhibit its growth and reproduction.

## Conclusion

In summary, spraying *B. coagulans* during incubation has no negative impact on hatchability while improving the percentage of healthy day-old chicks, promoting first-week growth performance. This treatment was also associated with reduced MS infection and alterations in cecal microbiota composition, including increased relative abundance of E. coprostanoligenes and *Negativibacillus*. These changes in microbial composition may contribute to enhanced host immune responses, as indicated by increased levels of IL-10, IgA, and IgG. These findings suggest that early-life exposure to *B. coagulans* via spraying may influence gut microbiota development and improve resistance to MS. Overall, the spray method offers an antibiotic-free alternative for controlling MS in chickens. Its technical advantages “operational simplicity and sustained efficacy” align closely with the demands of modern intensive farming systems.

## Funding

This research was funded by the National Key R & D Program of China (2024YFD200030403), Guizhou Provincial Key Technology R&D Program ([2023]004) and China Agricultural University–Jiajiang County People’s Government Professor Workstation for Poultry Breeding and Genetic Resources (202105510410408).

## CRediT authorship contribution statement

**Yimin Wei:** Writing – review & editing, Writing – original draft, Visualization, Validation, Methodology, Investigation, Formal analysis, Data curation. **Xuli Zhao:** Visualization, Validation, Methodology, Investigation, Formal analysis, Data curation. **Xing Chen:** Writing – original draft, Methodology, Investigation. **Xiaomeng Miao:** Resources, Project administration, Funding acquisition. **Wen Li:** Methodology, Investigation, Data curation. **Ying Ma:** Methodology, Investigation, Data curation. **Xiaoyu Zhao:** Resources, Project administration. **Zhonghua Ning:** Supervision, Resources, Project administration, Funding acquisition, Conceptualization.

## Disclosures

The authors declare that they have no known competing financial interests or personal relationships that could have appeared to influence the work reported in this paper.
